# Supplementary material for: Pancreatic circulating tumor cell profiling identifies LIN28B as a metastasis driver and drug target
Source: Nat Commun. 2020 Jul 3;11:3303. doi: 10.1038/s41467-020-17150-3 (PMC7335061; doi:10.1038/s41467-020-17150-3)
Supplement: Supplementary file 1 — Supplementary Information [file 41467_2020_17150_MOESM1_ESM.pdf]

SUPPLEMENTARY MATERIALS – Franses, Philipp et al. Pancreatic Circulating Tumor Cell Profiling Identifies LIN28B as a Metastasis Driver and Drug Target

Table of Contents:

- Supplementary materials and methods
- Tables S1-S6
- Figures S1-S11

Supplementary Materials and Methods

Cell Lines, RNAi, and Chemicals

Human pancreatic cancer cell lines Panc1, Panc0327, MiaPaca2, SUIT2, KP4, DAN-G, YAPC, BxPC3, SU86.86 were obtained from ATCC. Human pancreatic cancer cell line PDAC3 was developed in our laboratory and described previously[1]. All cell lines were grown in standard culture conditions and maintained as recommended by ATCC. No mycoplasma contamination has been detected in these lines.

For the production of replication-incompetent lentivirus, 293T cells were transfected with target plasmid, lentiviral gag/pol (Addgene plasmid #14887) and pMD2.G (Addgene plasmid #12259) using Lipofectamine 2000 (Thermo Fisher Scientific). Viral supernatants were harvested after 24 hours post-transfection, filtered and stored at -80°C. Cells were infected for 24 hours with 1 ml of the viral supernatant in presence of hexadimethrine bromide (8 µg/µL, Sigma). Infected cells were FACS-sorted for uniform, high GFP expression. CRISPR/Cas9 lentiviral plasmids targeting LIN28B exon 3.1, LIN28B exon 4 in and Ctrl1 plasmids as a control target, were a gift from the laboratory of Dr. George Daley (Harvard Medical School) and have been previously described[29]. The sequences for gRNAs are *LIN28B exon 3.1*: (CAC CGC AGA GCA AAC TAT TCA TGG A, AAA CTC CAT GAA TAG TTT GCT CTG C); *LIN28B exon 4*: (CAC CGC CTT GTA GAT GCT ACA ACT G, AAA CCA GTT GTA GCA TCT ACA AGG C); *Control 1*: (CAC CGG GTC CAT GGG TGG AGT TAC G, AAA CCG TAA CTC CAC CCA TGG ACC C). crLIN28Bex.3.1, crLIN28Bex4 and crCTRL1 lentiviruses were produced as described above. Puromycin was used for appropriate time using parental cells as controls to select transduced cells. The knockout of LIN28B expression was confirmed by western blot. GFP-Luciferase expressing cell lines were generated by lentiviral transduction with GFP/Luc vector (Addgene).

For transfection with siRNA, cells were transiently transfected using Dharmafect 1 reagent (Dharmacon) per manufacturer's instructions and either used in cellular assays or harvested for

RNA isolation 48 hours after transfection. All siRNAs were purchased from Dharmacon (ON-TARGET plus siRNA smartpools). The identifiers for all siRNA smartpools follow: NT siRNA(D-001810-10), GAPDH (D-001810-10), FN1 (L-009853-00), IGF2BP1 (D-001810-10), HMGA2 (D-001810-10).

For experiments utilizing transfection with let-7 microRNA sponge plasmid, we transfected either the baseline MSCV puro vector (Addgene plasmid #68469) or MSCV puro containing let-7 sponge (Addgene plasmid #29766) into target cells utilizing Lipofectamine 3000 (Thermo Fisher Scientific).

The chemical inhibitor of LIN28 was purchased from Tocris (cat #6068), cells were treated with 50 μM of inhibitor (dissolved in DMSO) in full media for three days. Control cells were treated with 0.1% DMSO as a vehicle control under otherwise equivalent conditions.

RNA in-situ hybridization (RNA-ISH)

Cell ISH was performed according to the ViewRNA ISH Cell Assay (Thermo Scientific) protocols. Briefly, cells were fixed in 4% formaldehyde for 30 min at room temperature, permeabilized by pretreating in buffer solution for 5 min at room temperature and digested with protease for 10 min. Target probe sets were applied and hybridized to the cells by incubating for 3 hours at 40°C. Probes were used at a dilution of 1:40 each for FN1 and SPARC (Type 1, Cy3 fluor) and 1:200 each for Type 6 (Cy5 fluor) keratin 7, 8, 18, and 19. Signal was amplified through the sequential hybridization of PreAmplifier and Amplifier mixes to the target probe set and target RNA molecules were detected by applying Label Probe Mix. Cells were then counterstained with DAPI (5 μg/ml, Life Technologies) and slides were mounted using ProLong Gold Antifade Reagent (Life Technologies). Fluorescence microscopy using a Nikon 90i was used to visualize target mRNA transcripts. Type 1 probes were detected in the Cy3 channel and Type 6 probes in the Cy5 channel.

Tissue RNA ISH was performed View RNA Tissue ISH (1-plex) technology using the manual platform (Catalogue No. 19931). FFPE tissue sections of human TMA were deparaffinized and pretreated to allow unmasking and RNA probe accessibility. This was done by first baking the slides at 60ºC for 1 hour followed by treatment with Histoclear and 100% Ethanol. The samples were then exposed to 1X Pretreatment Solution and Protease enzyme to allow probe accessibility. Hybridization was then performed at 40ºC where the LIN28B probe was incubated for 2 hours, to allow the target-specific probes to hybridize to target mRNA. This was preceded by a series of signal amplification steps; PreAmplifiers (PreAmps) were added to bind to the target-specific probe, and Amplifiers (Amps) were subsequently added to bind to the PreAmps. Next, type-specific label probes conjugated to alkaline phosphates were added to bind to the Amps, thus completing the branched DNA tree and providing signal amplification. The signal was visualized by addition of Fast Red substrate which binds to Type-1 label probe producing red precipitates. The target mRNAs were then scanned with an Aperio scanner.

These data were validated using a panel of ~10 resected PDAC FFPE specimens, with expected[2] variation in the degree of staining between and within samples.

Immunohistochemistry

Immunohistochemistry was performed on Leica BOND RX fully automated research stainer using ready to use Leica BOND Polymer Refine Detection kit (Catalog Number DS9800) containing a peroxide block, post primary, polymer reagent, DAB chromogen and hematoxylin counterstain. BOND Epitope Retrieval Solution 1 (Catalog Number AR9961), citrate based pH 6.0 epitope retrieval solution was used for heat-induced epitope retrieval (HIER) at 99-1000C, for 20 minutes. Primary antibodies used was IHCPlus™ Polyclonal LIN28B Antibody (Catalog Number LS-B3423-200), diluted to 10ug/ml with BOND Primary Antibody Diluent (AR9352), incubated on tissue at room temperature for 45 minutes.

These data were validated using a panel of ~10 resected PDAC FFPE specimens, with expected[2] variation in the degree of staining between and within samples.

Immunofluorescence staining and imaging

Immunostaining was performed by using a primary-secondary approach. Cells were cultured in chamber slides (Thermo Fisher). Next, cells were washed with PBS and fixed with 4% paraformaldehyde (Sigma) for 15 min, permeabilized with 0.2% Triton X-100 (Sigma), blocked with 10% goat serum in PBS for 60 min and stained primary antibodies overnight at 4°C. Cells were then washed with PBS-0.05%Tween-20 (Sigma) for 5 min each and stained with fluorescent secondary antibodies (Thermo Fisher) and nuclear 4,6-diamidino-2-phenylindole (DAPI) for 1 hour at room temperature. After rinsing the slides with PBS 3 times and cover slipping (Prolong Gold reagent, Thermo Fisher), they were stored at 4°C until image acquisition. Fluorescence images were acquired using a standard up-right fluorescent microscope (Nikon 90-I eclipse). The primary antibodies that were used were all validated by the vendors for immunofluorescent staining: FN1 (Santa Cruz Biotechnology, catalog #sc-8422, 1:50 dilution), HMGA2 (Cell Signaling Technology, catalog #8179, 1:200 dilution), LIN28B (Cell Signaling Technology, catalog #11965, 1:50 dilution), CK8/18 (Cell Signaling Technology, catalog #4546, 1:200 dilution), pan-keratin (Cell Signaling Technology, catalog #4545 1:500 dilution), CD45 (Cell Signaling Technology, catalog #55307, 1:500 dilution). DAPI (Thermo Fisher) was utilized at a concentration of 10 μg/mL. The secondary antibodies were all purchased from Thermo Fisher and were of goat origin and targeted toward the primary antibodies' species type, used at a dilution of 1:1000.

These data were replicated at least twice, with concordant results obtained for each replicate.

Proliferation and Migration Assays

Cell proliferation evaluation was performed with the PrestoBlue™ Cell Viability assay (Thermo Fisher Scientific). Cells ($2x10^3$) were plated in each well of a 96-well microplate. After 24 hours 5-volume-% resazurin reagent was added to each well, followed by a 1-hour incubation at 37°C. The fluorescence was measured at 590 nm.

To determine their migratory potential, cells were plated in a confluent monolayer for 24 hours. Using a 200-μL pipette tip, a straight scratch was created. After washing the cells with PBS, fresh media was added. The wound closure was monitored by taking images at 0 and 24 hours utilizing a phase contrast microscope at 4X magnification. Imagej v2 v2 was used to calculate the change of the cell-free area.

Sample sizes for these assays were chosen (4-6 replicates per group) based on past experience[3]. All attempts at replication of these data were successful, with expected variation in effect sizes but with similar order of magnitude differences in the experimental versus control conditions.

Anoikis and Invasion Assays

The spheroid invasion assay was performed as described elsewhere [4]. In brief, $5x10^3$ cells were plated in a 96-well ultra-low attachment round-bottom plates and incubated for 3 days. Afterwards, the medium was gently removed and Geltrex (Thermo Fisher) was added to coat the spheroids that have formed; after gel formation, fresh medium was added on top. Images were taken at day 0 and day 12. The invaded area was calculated by using Imagej v2.

For soft agar assays $1x10^4$ cells were resuspended in growth medium containing 0.3% noble agar and plated on a 12-well-plate that had previously been coated with 0.5% agar. After 3 weeks the colonies were fixed (4% PFA) and stained with 0.005% crystal violet (Sigma) solution. The colony number was counted by using Imagej v2.

Sample sizes for these assays were chosen (4-6 replicates per group) based on past experience[3]. All attempts at replication of these data were successful, with expected variation in

effect sizes but with similar order of magnitude differences in the experimental versus control conditions.

Western blot

To perform cell lysis for western blotting, cells were resuspended in lysis buffer (Cell extraction buffer, HALT protease inhibitor cocktail, PMSF (all from Thermo Fisher) incubated on ice for 15 minutes and cleared by centrifugation at 16,000 rpm for 15 minutes at 4 degrees C. Protein concentration was determined by BCA Protein Assay (Pierce). Proteins were resolved on 10% Bis–Tris gels (Thermo Fisher scientific), transferred to nitrocellulose membranes using iBot2 (ThermoFisher), and probed with the antibodies as recommended by the manufacturer. Chemiluminescence was detected with the Syngene G:Box camera (Synoptics). All analyses were performed with Imagej v2. The antibodies that were used were all application-specific validated antibodies from Cell Signaling Technology: LIN28B (catalog #11965, 1:1000 dilution), tubulin (catalog #2125, 1:5000 dilution), KRAS G12D (catalog #14429, 1:1000 dilution), HMGA2 (catalog #8179, 1:1000 dilution), GAPDH (catalog #5174, 1:5000 dilution). Secondary antibodies were also from Cell Signaling Technology: Anti-rabbit IgG HRP-conjugated (catalog #7054, 1:1000 dilution), Anti-mouse IgG HRP-conjgated (catalog #7076, 1:1000 dilution).

These data were replicated at least twice, and all attempts at replication were successful.

Patients and Blood Draws

Human blood for CTC analysis was obtained on two existing Dana-Farber Harvard Cancer Center (DFHCC) Institutional Review Boards protocols (05-300 and 18-179) at the Massachusetts General Hospital (MGH). Blood samples from healthy donors were obtained from anonymized discarded specimens collected at a blood donation center. Patients were consented and enrolled prior to blood draws. From 05-300, a maximum of 20 mL of blood was collected from a total of 56 subjects, of which 21 were healthy donors, 17 had localized PDAC, and 18 had metastatic PDAC.

Patient cohorts and clinical characteristics are provided in Tables 1 and 2 and Supplementary Table 1. From 18-179, the blood draw parameters were the same, and the patients all had localized PDAC; since this trial remains ongoing, we did not have access to clinical and/or outcome data from these patients for the purposes of the current publication. Patient allocation to each the groups noted in our work was not random and was defined by their disease state. Blinding during collection and analysis was not performed, as knowledge of each "type" of patient (HD, locPDAC, metPDAC) group was necessary for the analysis.

RT-qPCR

Measurement of microRNA and mRNA expression levels were done by qRT-PCR on an Applied Biosystems AB7500 Real Time PCR System. For microRNA analyses the miRCURY LNA miRNA PCR Assays (Qiagen) were used and mRNA expression levels were measured by using SYBR Green PowerUP master mix (Thermo Fisher). Ct values were normalized to SNORD44 for microRNA and GAPDH for mRNA, respectively, and relative changes were calculated using the 2-ΔΔCt method.

mRNA primers:

LIN28B (fwd: GAG TCA ATA CGG GTA ACA GGA C, rev: CAC CAC AGT TGT AGC ATC TAT CT)

IGF2BP1 (fwd: CAG TCC AAG ATA GAC GTG CAT AG, rev: CTC AGG GTT GTA AAG GGT AAG G),

HMGA2 (fwd: CTG CTC AGG AGG AAA CTG AAG, rev: CAC TAA ACC TGG GAC TGT GAA G)

FN1 (fwd: GCT CAA CGA GAA CCG TAT CCG, rev: TGA GGT ACG TGA GGT TGC CA)

SPARC (fwd: CGT CCT GGT CAC CCT GTA TG, rev: GGG AGA GGT ACC CGT CAA TG)

GAPDH (fwd: ACA GTC AGC CGC ATC TTC TT, rev: TGG AAG ATG GTG ATG GGA TT)

miRNA PCR assays: All were purchased from Qiagen utilizing their pre-designed miRCURY LNA PCR assays targeting mature miRNAs (catalog #339306) designed with primers oriented in the

5p direction. let-7a (YP00205727), let-7b (YP00204750), let-7c (339306), let-7d (YP00204124), let-7e (YP00205301), let-7f (YP00204359), let-7g (YP00204565), let-7i (YP00204394), miR-98 (YP00204640), and SNORD44 (YP00203092) were used.

Animal Models

All animal experiments and animal care were performed according to institutional guidelines at Massachusetts General Hospital (MGH) and approved by the animal protocol (2014N000321). Animals were euthanized as per animal protocol guidelines. Six- to ten-week old, female, NSG (NSG; NOD.Cg-Prkdcscid Il2rgtm1Wjl/Sz) mice were purchased from Jackson Laboratories. The mice were housed in cages of no more than 4 mice/cage at a temperature of 23+/- 3 degree C and relative humidity of 30-70% with 14 hr / 10 hr light/dark cycle.

Tail Vein Assay

2.5 x 10^5 GFP-luciferase tagged PANC1 cells (crLIN28Bex3.1, crLIN28BCTRL1) in 100 µl PBS were injected into the tail vein of NSG mice; n=8 per experimental group. The Bioluminescence signal was monitored weekly using in vivo luciferase imaging on the IVIS Lumina platform (Perkin Elmer/Caliper) after iv-injection of 100 µL 30 mM D-Luciferin (Thermo Fisher). The total photon count in the region of interest was quantified using the ROI function of the software and IVIS luminescence signal was normalized to photon (p s−1 cm−2 sr−1).

Orthotopic Xenograft Assay

Orthotopic Implantation Assay

GFP-luciferase tagged PANC1 cells were implanted orthotopically into the pancreas (crLIN28Bex3.1, crLIN28Bctrl1); n=8 per experimental group. Briefly, mice were anesthetized with isoflurane, hair removal was performed, and lubricant was applied into the eyes of the mice. The abdominal wall was sterilized with 10% povidone iodine solution and 70% ethanol. Using sterile surgical instruments, a small incision left lateral from the midline was performed. After locating

the pancreas, 30µl with 2×10^{5} of the cell-Matrigel suspensions (serum-free OptiMEM, Thermo Fisher, and Matrigel (Corning, growth-factor reduced), 1:1) were introduced into the pancreas. The peritoneum was closed using absorbable sutures (4-0 DemeGUT™) and the skin was sealed using silk (4-0 LOOK 780B). The tumour burden was monitored weekly by measuring the Bioluminescence as described above. After 8 weeks, a cardiocentesis was performed to collect blood. Therefore, mice were anesthetized with isoflurane, the chest wall was sterilized with 70% ethanol and the chest soft tissue was blunt-dissected. Using a 25G needle, ~1 ml of blood was drawn via cardiac puncture. Syringes and collecting tubes were previously primed with EDTA (10 nM). Next, liver, lungs and primary tumour were harvested and separately imaged for luminescence. Mouse tissue was then flash frozen with liquid nitrogen and formalin-fixed for IHC. Tissues were stored at -80°C.

Public Dataset Access

The data utilized for RNA-seq gene expression from normal tissues (Supplementary Fig. 1c) were obtained from the GTEx Portal on 12/31/2019. The data utilized for RNA-seq gene expression analysis of TCGA pancreatic cancer data were obtained from Cbioportal[5, 6] in February 2020. miRTarbase[7] was used for miRNA interaction target prediction.

Supplementary Tables and Figures

ID	Sex	Age
HD01	M	28
HD02	M	39
HD03	F	51
HD04	F	53
HD05	F	44
HD06	M	57
HD07	M	62
HD08	M	67
HD09	M	23
HD10	F	27
HD11	F	27
HD12	F	43
HD13	F	52
HD14	F	56
HD15	F	62
HD16	F	70
HD17	F	70
HD18	M	40
HD19	M	46
HD20	M	53
HD21	M	55

Supplementary Table 1. Healthy donor (HD) characteristics at the time of blood draw for CTC purification.

Genes upregulated in locPDAC CTCs vs HD (FDR<0.1)		Genes upregulated in metPDAC CTCs vs HD (FDR<0.1)	
Gene	Log2(FC)	Gene	Log2(FC)
RP11-126O2	3.94589158	MUC6	7.6995057
RP11-504A1	3.8240122	TMEM184A	6.99696583
RP11-363G1	3.81765766	SLC6A19	6.86766989
MBNL3	3.80826228	RAB3IL1	6.81527135
MT-TP	3.79076543	AGRN	6.76590768
SPIN4-AS1	3.71716761	PLA2G2F	6.69950917
AC000099.1	3.71282476	MUC2	6.65885222
FAM35CP	3.712086	GPR123-AS1	6.63475965
PARM1	3.65037261	HHIPL1	6.59351044
HEPACAM2	3.63081479	VWA1	6.57924719
RP3-428A13	3.59963616	PTPN5	6.57795013
MUC3A	3.55424136	ELFN2	6.54101214
CHAC2	3.51340579	AC027601.1	6.50439311
WNK1	3.4991149	SYT8	6.49574473
EPS15P1	3.49832146	DUX4L19	6.46581794
RPTN	3.4763622	LYNX1	6.45520811
SGCE	3.46486876	MAGEL2	6.43719718
CHST9	3.39073332	FLJ16779	6.38026692
RP11-346C2	3.38209413	FAM83H	6.3552723
RP11-304C1	3.3816941	DIO3OS	6.29507641
RP11-483C6	3.3104704	TTBK1	6.29025316
LRRN1	3.30952454	ARHGEF16	6.28597002
CLUL1	3.30918847	AC139099.7	6.23176585
PDC	3.2295439	LRRC3	6.22937681
CTD-2076M1	3.21143829	CFAP46	6.20383609
MKI67	3.20987091	CITF22-49D	6.17947442
RP11-1299A	3.20740149	CEP170B	6.14273228
RP11-1144P	3.20401727	KIF26A	6.13926537
NANOGP11	3.20179392	KCNQ2	6.13894899
MAP9	3.19620843	NTSR1	6.1382543
PARPBP	3.19530528	RTN4R	6.13651119
RP11-145P1	3.19373177	SLC6A9	6.13128591
IL33	3.17909668	AC007326.1	6.12804246
MBNL1-AS1	3.15065609	DDN	6.11791859
GJA1	3.14873022	LINC00982	6.11595729
NUF2	3.10237858	ARHGEF17	6.10351974
RP11-16E18	3.08826012	CHST3	6.09056029
RP11-907D1	3.07981931	BCAR1	6.08749888
AADACL3	3.06560761	HCN4	6.08117963
ZSWIM2	3.0506853	BSN	6.07819195
LINC01320	3.04240002	ESPNL	6.05792133
LINC01297	3.03444614	NXPH3	6.0550365
TMTC1	3.03435078	LINC01166	6.03863487
LINC01579	3.01819642	GPC1	6.03498472
SMYD1	3.01263982	RP11-54O7.	6.03098395
RP11-203B7	3.01129379	GPR78	5.99740706
PGAP1	3.01002449	RIMS4	5.99094449
HMGN5	3.00954586	GRK1	5.98486877
TMIGD1	3.00760811	HRNR	5.96902915
OR12D2	2.99489376	PPP1R3G	5.96398998
STARD4-AS	2.98022998	GPRIN2	5.95762715
GOLIM4	2.9769132	XKR7	5.95515806
RBM46	2.96989954	SMIM1	5.95370228
AC093590.1	2.96879083	ARHGAP23	5.95104359
CPOX	2.96752001	ADAM33	5.94356435
CTA-392E5.1	2.96728171	NFIX	5.93091503
CHRM2	2.95715148	CECR6	5.92841746
TSPAN7	2.94701637	NHLRC4	5.92431613
SPRED1	2.9348835	FLNC	5.90009288
MT-TI	2.9329205	WFIKKN1	5.89275229
SLC2A1	2.92725773	DUX4L18	5.89025746
NFIX	2.92133475	TPRN	5.88315612
KIF11	2.914578	RP11-503N1	5.88231794
DSPP	2.90287	AHNAK2	5.86797351
RP11-260M2	2.8996819	TMEM132A	5.86716407
RP11-159D1	2.89672856	RP4-734G22	5.86503582
RP11-717H1	2.89505842	FAM181A	5.86362083
SNX16	2.8948321	HGFAC	5.85641411
GYPA	2.89303325	CHRNA4	5.85590663
SLITRK2	2.89205081	LRFN3	5.85072033
RP11-467L1	2.88869929	FLG2	5.84601247
PABPC4L	2.87788271	TEX22	5.84473128
YOD1	2.87197623	CRB2	5.83999669
DEPDC1-AS	2.86516689	XIRP1	5.8283299
MAEL	2.85955439	MUC5B	5.825505
COL3A1	2.85546585	RP13-580B1	5.81693412
MUC16	2.85446627	RP11-958N2	5.80722607
VTCN1	2.85168453	CROCCP1	5.80493387
TMEM67	2.85040614	PNCK	5.79059147
AC064875.2	2.84711867	C6orf223	5.78738772
ERICH3	2.84188141	HOXC12	5.78697009
KHDRBS2	2.83925	ZFP92	5.78319921
CTD-2123J1	2.83696796	CYP26B1	5.77607128
ACSL6	2.83483592	AIRE	5.77184044
RP11-30P6.	2.83296166	LINC01168	5.76068415
CENPE	2.83082657	RP11-126O2	5.75102823
TRIM36-IT1	2.82126534	B4GALNT1	5.736375
LMCD1-AS1	2.82066705	RP5-908M14	5.73330376
RP11-643G5	2.80340206	YBX2	5.73297727
OMD	2.80267064	DAGLA	5.72855146
MAOA	2.79990952	SSPO	5.72695539
ACTBL2	2.7970771	EMX1	5.72652523
RGAG1	2.79488271	FEM1AP4	5.72077741
PDE1A	2.79239833	IGSF9	5.72044273
PLVAP	2.78814279	MMP24	5.71857727
SNAPC1	2.77965573	RP13-870H1	5.70942187
LRRC53	2.77210454	BEGAIN	5.70798404
METTL25	2.7706392	EPHA8	5.69742589
RP11-925D8	2.76700908	AHSP	5.6932512

Supplementary Table 2. Top 100 differentially expressed genes (FDR<0.10) for locPDAC CTCs, and PDACmet CTCs versus blood cells purified from HD controls.

**LIN28B correlates**		**WNT5A correlates**		**LGALS3 correlates**		**KLF4 correlates**	
**Gene**	**Correlation coeff**	**Gene**	**Correlation coeff**	**Gene**	**Correlation coeff**	**Gene**	**Correlation coeff**
GPR112	0.823	RANBP17	0.908	BPGM	0.926	TSHZ1	0.794
ATP6AP1L	0.782	PDE1A	0.906	CA1	0.901	SIPA1L3	0.767
RP11-752L20.3	0.777	RPS6KA6	0.901	MKRN1	0.896	ZNF740	0.752
PHACTR3	0.770	PCDH9	0.897	BTF3	0.884	SKIL	0.745
KIAA2022	0.759	NEBL	0.890	EIF1B	0.876	HIVEP3	0.742
CHST9	0.751	SLC12A2	0.889	BNIP3L	0.871	SPACA6P-AS	0.742
FAM71F2	0.750	SNAP25-AS1	0.889	CAT	0.861	TRERF1	0.742
RIMS1	0.748	RP11-358D17.2	0.878	STRADB	0.861	CDKN1C	0.737
SARM1	0.744	PCDHA4	0.878	MPP1	0.858	LINC00661	0.727
ADH1B	0.744	KCTD16	0.878	PSMF1	0.858	ARHGEF10	0.721
RP11-775C24.3	0.744	ZNF704	0.877	RIOK3	0.852	GAS6	0.719
TAF1L	0.743	HOMER1	0.877	TOP1	0.852	DLL4	0.701
FCRL5	0.742	ARID1B	0.876	ISCA1	0.842	USP43	0.700
CNTNAP5	0.741	ZNF257	0.876	THOC7	0.840	MESDC1	0.698
RP11-93G23.2	0.739	LIMCH1	0.875	SEC62	0.838	SHOX2	0.695
STEAP2	0.739	TSHZ3	0.874	GABARAPL2	0.838	SNX18	0.694
MORC4	0.738	FAM221B	0.874	PRDX6	0.834	RSBN1	0.689
BAI3	0.735	DLG2	0.874	TRAK2	0.832	CYTH3	0.688
RP11-475J5.4	0.730	TSC22D2	0.873	HBD	0.832	THBD	0.679
B3GALT5	0.729	DSC2	0.873	RBX1	0.824	TSPYL4	0.678
RP1-274L7.4	0.728	RGS17	0.872	UBB	0.824	PGRMC2	0.673
MAPK10	0.726	GRID2	0.871	RPS12	0.821	FAM229A	0.671
GALNT13	0.726	DST	0.871	SNCA	0.817	ZNF273	0.670
CTB-113D17.1	0.723	Y_RNA	0.870	DCAF12	0.817	KLHL28	0.670
OPN5	0.722	LINC01579	0.870	OPTN	0.812	ZNF319	0.664
RP11-760D2.5	0.722	COL19A1	0.869	SELK	0.809	TMEM229A	0.662
ZNF619	0.722	HDGFRP3	0.869	ATP5E	0.801	HDGFRP3	0.661
XKR4	0.719	GPR137C	0.868	RNF10	0.801	RP11-598F7.4	0.656
ERVW-1	0.716	LINC00971	0.868	CTSB	0.801	TENM3	0.655
ZNF334	0.716	FGF13	0.867	TPGS2	0.798	BEND3	0.654
RP11-359E3.4	0.715	NRXN1	0.866	FECH	0.791	PIK3CG	0.653
LL22NC03-22D1.1	0.713	CCDC150	0.865	MXI1	0.789	KCNT2	0.650
ZNF615	0.713	CLUL1	0.864	HBG1	0.787	PCDHA4	0.649
MTND1P36	0.711	SUMO4	0.863	FBXO9	0.787	CTD-3187F8.14	0.646
LINC00347	0.710	MAGI1	0.862	ST13	0.787	CDH5	0.644
ANK2	0.710	BMP3	0.861	SKP1	0.785	LDLRAD4	0.643
KANK1	0.709	SYT14	0.860	GSPT1	0.783	EFNB2	0.642
DCLK1	0.708	PGGT1B	0.860	ANP32B	0.782	ZBED6CL	0.641
EPB41L4B	0.707	FAM92A1P2	0.859	ZMAT2	0.780	KCTD12	0.640
TRIO	0.706	SOX11	0.859	PCGF5	0.778	ESYT3	0.639
TRPC5	0.705	NAALADL2	0.859	SOD1	0.777	COL9A1	0.637
TMEM132B	0.704	GAN	0.858	RPIA	0.776	RP1	0.637
RP1-101G11.3	0.704	TMTC1	0.858	GMPR	0.774	FEZF1	0.636
CSRNP3	0.703	RNASEH2B-AS1	0.857	FAM46C	0.771	REM2	0.634
CTA-544A11.1	0.701	ST6GALNAC3	0.856	EIF3J	0.767	PIGP	0.632
CTD-2313J23.1	0.700	MTTP	0.855	CCNDBP1	0.766	ZIC5	0.632
SLC12A1	0.700	RP11-346C20.3	0.855	GYPA	0.763	ZSWIM4	0.629
ALMS1	0.699	NF1	0.854	FGFR1OP2	0.762	RHOB	0.628
OPCML	0.699	ZNF333	0.853	HBB	0.761	RANBP3	0.628
AC105921.5	0.699	RBM46	0.853	MAP2K3	0.757	DHX33	0.628
RP11-268G12.1	0.698	ESR1	0.851	FAM210B	0.753	SOX9	0.628
CFTR	0.696	TMEM67	0.851	DCAF6	0.747	MAML1	0.628
SLCO5A1	0.695	DGKE	0.851	FBXO7	0.744	BCL7A	0.628
CALML3-AS1	0.694	ZNF462	0.851	UBBP4	0.743	MECOM	0.627
AC006548.28	0.693	RP11-976B16.1	0.851	PIP4K2A	0.740	ZC3H4	0.627
NTRK3	0.693	SCAI	0.850	HBG2	0.739	AUTS2	0.623
SYT16	0.693	GULP1	0.849	SELENBP1	0.738	RP11-357P18.2	0.622
LINC00900	0.690	ABCC9	0.849	PITHD1	0.736	IGFBP3	0.622
ST8SIA3	0.689	PGAP1	0.848	AC104389.1	0.736	PHKA1	0.621
IMPG2	0.689	CHL1	0.848	IFIT1B	0.733	NR2F2	0.621
KIRREL3	0.689	KCNQ1OT1	0.847	CKS2	0.731	HOXA3	0.620
AC083843.1	0.688	NPY1R	0.847	TERF2IP	0.730	DCHS2	0.619
HECW2	0.688	ALS2CR11	0.847	NDUFC1	0.730	SOX11	0.619
GRIN2B	0.686	CXXC4	0.847	YBX1	0.729	AFF3	0.619
RP11-508N22.12	0.686	BMPR2	0.846	AHSP	0.728	MNT	0.619
FEZ2	0.686	ENAH	0.845	CCDC176	0.725	CSMD1	0.618
IL17RE	0.685	ERBB4	0.845	UBE2B	0.725	ABCB10P1	0.618
RTKN2	0.684	EVI5	0.844	HEMGN	0.724	CLUL1	0.617
GABRB3	0.684	ACVR1C	0.844	GYPB	0.719	LDB3	0.617
NPR3	0.681	SYCP2	0.843	CCNI	0.719	HES1	0.616
KCNQ3	0.680	NAALAD2	0.843	RAB2B	0.717	MCAM	0.615
RP11-281P23.1	0.680	LRRN1	0.843	SLC2A1	0.717	MANEA-AS1	0.615
CACHD1	0.679	PCCA	0.842	LINC00570	0.717	SALL3	0.615
RP11-295P9.3	0.679	ONECUT2	0.842	CISD2	0.716	TTC28	0.614
RP11-983P16.4	0.678	LRAT	0.842	MMADHC	0.715	ZCCHC3	0.613
RP11-766F14.2	0.678	RBM26-AS1	0.841	TCEA1	0.714	METTL10	0.613
RP11-379F4.4	0.678	KBTBD7	0.841	RP11-85G21.3	0.711	TMEM67	0.613
PRLR	0.678	SLITRK5	0.841	SLC25A38	0.710	IRX2	0.612
SLC1A3	0.677	APTR	0.840	CR1L	0.710	EXOSC7	0.612
RP3-405J10.3	0.675	ANKRD31	0.839	MBNL3	0.708	LHX2	0.611
DRP2	0.675	ABCA10	0.839	NAP1L4	0.707	GLI3	0.611
FUT9	0.675	GALNTL6	0.838	CMAS	0.703	CCDC7	0.610
RP11-39H3.2	0.675	RP11-63B13.1	0.838	RHD	0.700	WASF3	0.610
RP11-710C12.1	0.675	PIK3C3	0.838	UBAP1	0.699	PBX3	0.608
RP11-638L3.1	0.675	COX18	0.838	OAZ1	0.699	FAM46A	0.607
OSBPL10	0.674	SOX2-OT	0.837	FAM104A	0.697	DISP2	0.607
NME9	0.674	RGS5	0.837	KRT1	0.693	RGS17	0.607
LAMA4	0.672	IRAK1BP1	0.836	DCUN1D1	0.692	AC133785.1	0.607
AC105342.1	0.671	ZFP30	0.836	FAU	0.692	FAM92A1P2	0.605
OMG	0.670	KIF21A	0.834	OPA1	0.687	MTUS2	0.603
DTNA	0.670	ADAM22	0.834	DHX29	0.687	RP3-466P17.1	0.602
HECW1	0.670	KIAA1377	0.834	RALBP1	0.685	NDRG1	0.601
ZNF501	0.668	SMURF2	0.833	SLC25A39	0.684	IRS1	0.601
CTC-529P8.1	0.667	RP11-2E11.6	0.832	RPL38	0.682	SREBF2	0.601
AC093642.1	0.667	SCN11A	0.832	ARL4A	0.681	GRB7	0.601
AC074286.1	0.666	FLRT2	0.832	COPZ1	0.680	TWIST1	0.600
MAL2	0.666	ZBTB38	0.831	BLVRB	0.679	COL6A4P1	0.600
GLI1	0.665	FAM171B	0.831	OSBP2	0.678	RP11-976B16.1	0.599
RP11-113I24.1	0.664	NHSL1	0.831	HBM	0.678	MUM1	0.599
ACVR2B	0.664	EYA4	0.830	ISCU	0.675	ARID1B	0.599

Supplementary Table 3. Top 100 Genes correlated with LIN28B, WNT5A, LGALS3, and KLF4 in PDAC CTCs. Pearson correlation coefficient shown with each gene.

		TIME OF BLOOD DRAW			TIME OF SURVIVAL ANALYSIS		CTC RNA-SEQ GENE EXPRESSION VALUES (RPM)				
**ID**	**Sex**	**Age**	**CA19-9 (U/mL)**	**CEA (ng/mL)**	**Alive at analysis?**	**OS (days)**	**LIN28B**	**HMGA2**	**KLF4**	**WNT5A**	**LGALS3**
locPDAC01	M	82	224	3.6	N	601	18.2	20.8	0.0	262.5	57.2
locPDAC02	F	49	566	1.1	N	317	26.0	23.4	7.8	199.8	33.7
locPDAC03	M	70	40	4.7	N	208	0.0	0.0	0.0	4744.2	1.7
locPDAC04	M	72	82	5	N	1139	0.0	26.3	105.2	65.7	52.6
locPDAC05	M	65	1943	4.8	N	1016	8.3	16.7	33.4	191.8	8.3
locPDAC06	F	74	289	2.5	N	524	0.0	4.6	6.9	169.5	9.2
locPDAC07	F	59	418	2.9	N	1192	2.0	15.6	5.9	1527.3	5.9
locPDAC08	M	61	179	2	N	1090	20.7	13.8	13.8	124.4	41.5
locPDAC09	M	60	132	3.2	N	348	20.7	48.4	48.4	44.9	72.6
locPDAC10	F	89	11.7	24	**	**	16.9	25.3	6.3	14.7	63.2
locPDAC11	F	50	1040	8.3	N	1298	0.0	52.1	20.8	166.7	198.0
locPDAC12	F	66	2	1.5	**Y**	1306	5.2	20.9	15.7	149.2	68.0
locPDAC13	F	83	19	1.5	N	1001	23.0	7.7	199.2	76.6	7.7
locPDAC14	M	69	63	4.9	**Y**	1366	0.0	0.0	313.4	1334.6	5.1
locPDAC15	F	69	369	5.4	N	219	0.0	23.8	428.6	131.0	190.5
locPDAC16	M	76	15	3.2	N	1149	9.5	4.7	114.0	180.5	76.0
locPDAC17	F	75	70	2.1	N	1254	5.2	5.2	367.2	813.2	10.5
metPDAC01	M	57	7	1.1	N	2274	0.0	0.0	11.0	5.5	296.7
metPDAC02	F	49	117	4.8	N	1434	0.0	0.0	0.0	0.0	6411.5
metPDAC03	F	53	706	5.5	N	1974	0.0	0.0	1.5	1.5	60.8
metPDAC04	F	58	1307	54	N	750	23.5	3.9	15.7	3.9	27.4
metPDAC05	F	66	7268	2.6	N	503	0.0	60.6	69.3	14.4	0.0
metPDAC06	M	55	4	-	N	725	0.0	1.9	15.5	0.0	282.7
metPDAC07	M	60	354	2.9	N	564	0.7	8.0	15.4	0.0	789.7
metPDAC08	M	66	355	3.4	N	1016	0.0	0.0	0.0	0.0	1606.8
metPDAC09	F	70	18	1.5	Y	1825	0.0	0.0	0.0	2.5	3853.8
metPDAC10	F	75	<1	11	N	1319	0.0	0.0	0.0	0.0	395.2
metPDAC11	M	49	32	6.4	Y	1440	0.0	0.0	8.7	1.4	1045.1
metPDAC12	M	66	174	19	N	1410	0.0	6.0	5.1	0.0	2078.5
metPDAC13	F	71	39	5	N	1166	11.2	73.0	89.8	50.5	11.2
metPDAC14	F	70	54	9.9	N	1422	0.0	0.0	0.0	3.9	420.8
metPDAC15	F	71	49	1.6	N	2040	22.5	78.6	56.1	84.2	5.6
metPDAC16	F	79	19	-	Y	1271	9.9	44.4	14.8	39.5	9.9
metPDAC17	M	67	593	4.2	Y	436	116.3	0.0	0.0	29.1	0.0
metPDAC18	F	68	39	2	N	121	37.9	15.2	7.6	30.3	60.6

** Lost to follow-up immediately after discharge from hospital after surgical resection. Not included in survival analysis.

Supplementary Table 4. Survival data – along with other circulating biomarkers – for the locPDAC and metPDAC cohorts. Survival data censored on 10/15/2019.

ID	POS/NEG	ID	POS/NEG	ID	POS/NEG	ID	POS/NEG
locPDAC_B_01	neg	locPDAC_B_21	pos	locPDAC_B_41	neg	locPDAC_B_61	neg
locPDAC_B_02	neg	locPDAC_B_22	neg	locPDAC_B_42	pos	locPDAC_B_62	neg
locPDAC_B_03	neg	locPDAC_B_23	neg	locPDAC_B_43	pos	locPDAC_B_63	neg
locPDAC_B_04	neg	locPDAC_B_24	neg	locPDAC_B_44	pos	locPDAC_B_64	neg
locPDAC_B_05	neg	locPDAC_B_25	pos	locPDAC_B_45	pos	locPDAC_B_65	neg
locPDAC_B_06	neg	locPDAC_B_26	pos	locPDAC_B_46	pos	locPDAC_B_66	neg
locPDAC_B_07	pos	locPDAC_B_27	pos	locPDAC_B_47	pos	locPDAC_B_67	neg
locPDAC_B_08	pos	locPDAC_B_28	neg	locPDAC_B_48	neg	locPDAC_B_68	neg
locPDAC_B_09	neg	locPDAC_B_29	neg	locPDAC_B_49	pos	locPDAC_B_69	neg
locPDAC_B_10	neg	locPDAC_B_30	pos	locPDAC_B_50	pos	locPDAC_B_70	pos
locPDAC_B_11	neg	locPDAC_B_31	pos	locPDAC_B_51	neg	locPDAC_B_71	neg
locPDAC_B_12	pos	locPDAC_B_32	pos	locPDAC_B_52	neg	locPDAC_B_72	neg
locPDAC_B_13	pos	locPDAC_B_33	pos	locPDAC_B_53	pos	locPDAC_B_73	neg
locPDAC_B_14	pos	locPDAC_B_34	neg	locPDAC_B_54	neg	locPDAC_B_74	neg
locPDAC_B_15	pos	locPDAC_B_35	neg	locPDAC_B_55	neg	locPDAC_B_75	neg
locPDAC_B_16	pos	locPDAC_B_36	neg	locPDAC_B_56	pos	locPDAC_B_76	neg
locPDAC_B_17	neg	locPDAC_B_37	pos	locPDAC_B_57	neg	locPDAC_B_77	neg
locPDAC_B_18	neg	locPDAC_B_38	pos	locPDAC_B_58	neg	locPDAC_B_78	neg
locPDAC_B_19	pos	locPDAC_B_39	pos	locPDAC_B_59	neg	locPDAC_B_79	neg
locPDAC_B_20	pos	locPDAC_B_40	pos	locPDAC_B_60	neg	locPDAC_B_80	neg

Supplementary Table 5. RNA ISH scoring (positive or negative) for resectable pancreatic cancer tissue microarrays.

A

Gene	log2(FC)
MMP1	1.73
PRSS3	1.12
HMOX1	1.12
ATG101	1.10
SQSTM1	1.10
LAMP3	1.07
ZFPL1	1.06
PMP22	1.01
RANGRF	0.99
HTR7	0.97
CLGN	0.97
DDIT3	0.97
BRF2	0.94
TXNIP	0.93
ADPRHL1	0.92
MAZ	0.92
ZNF622	0.92
IFT46	0.91
SIAH1	0.91
LRRC8E	0.90
CPVL	0.90
XRCC3	0.89
MRPS18A	0.89
GOSR2	0.88
TSPAN1	0.88
TIPARP	0.87
RNMTL1	0.87
RHCG	0.87
ZSCAN21	0.86
FOXD1	0.86
SDR39U1	0.86
TXNL4B	0.86
SH3BP5	0.85
SLC41A2	0.85
DNASE1L1	0.85
FAM219B	0.85
MAFF	0.84
BCL2L1	0.84
KLHL21	0.84
WDR25	0.84
HEXA	0.84
SARM1	0.83
ID2	0.83
IL1RL1	0.83
CEBPB	0.81
FOSL2	0.81
PPP1R15A	0.80
GPAT3	0.80
WSCD1	0.79
AFTPH	0.79

B

Gene	-log2(FC)
LIN28B	1.17
BMF	1.02
SHISA3	1.02
COL21A1	0.95
HMGA2	0.95
ALPPL2	0.95
RGS17	0.93
CPED1	0.93
TTN	0.92
UNC5C	0.90
PBX2	0.86
NID1	0.85
GABBR1	0.85
SPARC	0.84
ARID3B	0.84
FABP4	0.84
ZNF814	0.82
SV2A	0.82
MDC1	0.79
MT-CO3	0.79
BCAS1	0.78
SLC29A2	0.77
PDE8B	0.76
MARCKS	0.76
GRHL3	0.75
HNRNPA3	0.75
ADAMTS3	0.75
MT-CO2	0.74
MT-ND3	0.74
SCUBE3	0.73
COL1A1	0.73
KIF26B	0.72
ZC3HAV1L	0.72
VAMP2	0.71
EP400	0.71
DICER1	0.71
F2RL2	0.71
TRPM8	0.71
SLCO4C1	0.71
FUS	0.71
TFCP2L1	0.70
RP5-862P8	0.70
FASN	0.70
MT-CO1	0.70
GUCY1A2	0.69
SCNN1A	0.69
FAM98B	0.69
PLAGL2	0.69
ANP32B	0.69
MT-ND6	0.68

Supplementary Table 6. Top 50 upregulated (A) and downregulated (B) genes in PANC1 LIN28B CRISPR knockout cells relative to nonsense gRNA-transduced control cells.

a

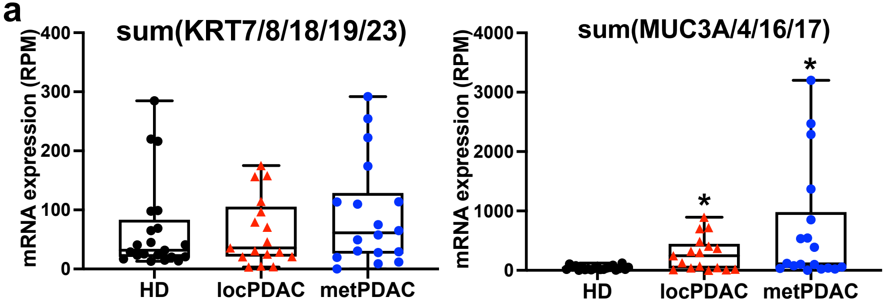

b

Gene	whole blood median RPM	pancreas median RPM	highest median RPM	ID of highest median RPM site
MUC3A	0.0087	0.51	48.6	small intestine
MUC4	0.010	0.069	24.7	transverse colon
MUC16	0.0046	0.0058	4.0	minor salivary gland
MUC17	0.000	0.0019	28.1	small intestine - terminal ileum

c d

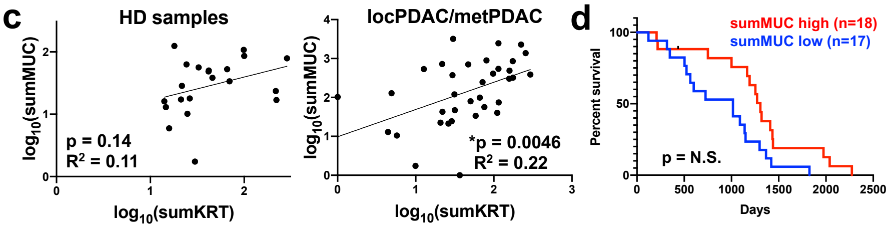

e

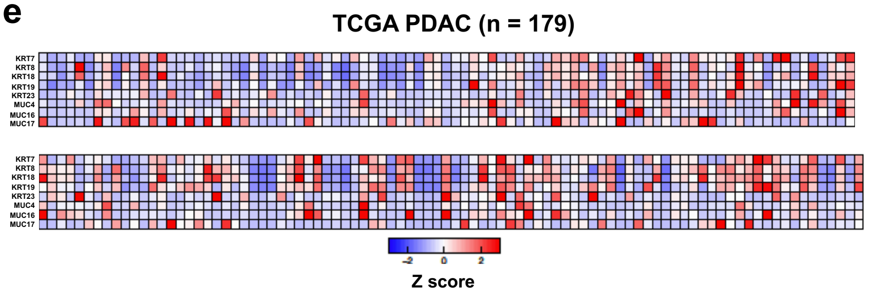

Supplementary Figure 1. Mucin mRNA expression is more specific than keratin mRNA expression for CTCs purified from patients with localized PDAC or metastatic PDAC. (**a**) Box plots showing the sum of KRT and MUC gene expression in CTCs purified from patients with localized PDAC and metastatic PDAC compared with control blood processed from healthy donor (HD) volunteers. * FDR<0.10 in differential expression analysis of locPDAC (n = 17) vs HD (n = 21) or metPDAC (n = 18) vs HD (n = 21). (**b**) Gene expression (RPM) of mucin genes in normal tissues as generated by the GTEX portal. (**c**) Log-log plot of the sum of RPM-normalized mucin (*MUC3A/4/16/17*) versus the sum of keratins (*KRT7/8/18/19/23*) in HDs (left) and CTCs purified from the blood of patients with localized PDAC and metastatic PDAC. * p = 0.005, least squares linear regression for HD samples (n = 21) or locPDAC (n=17) plus metPDAC (n=18), no multiple-test adjustments were made. (**d**) Survival in our patient cohort of patients with sum of MUC expression higher than the median versus sum of MUC expression lower than the median. p > 0.05 by Gehan-Breslow-Wilcoxon test. (**e**) Heatmap of TCGA bulk RNA sequencing Z score-

normalized data (Cbioportal[5, 6]) from resected PDAC tissue showing heterogeneous expression of KRT and MUC genes.

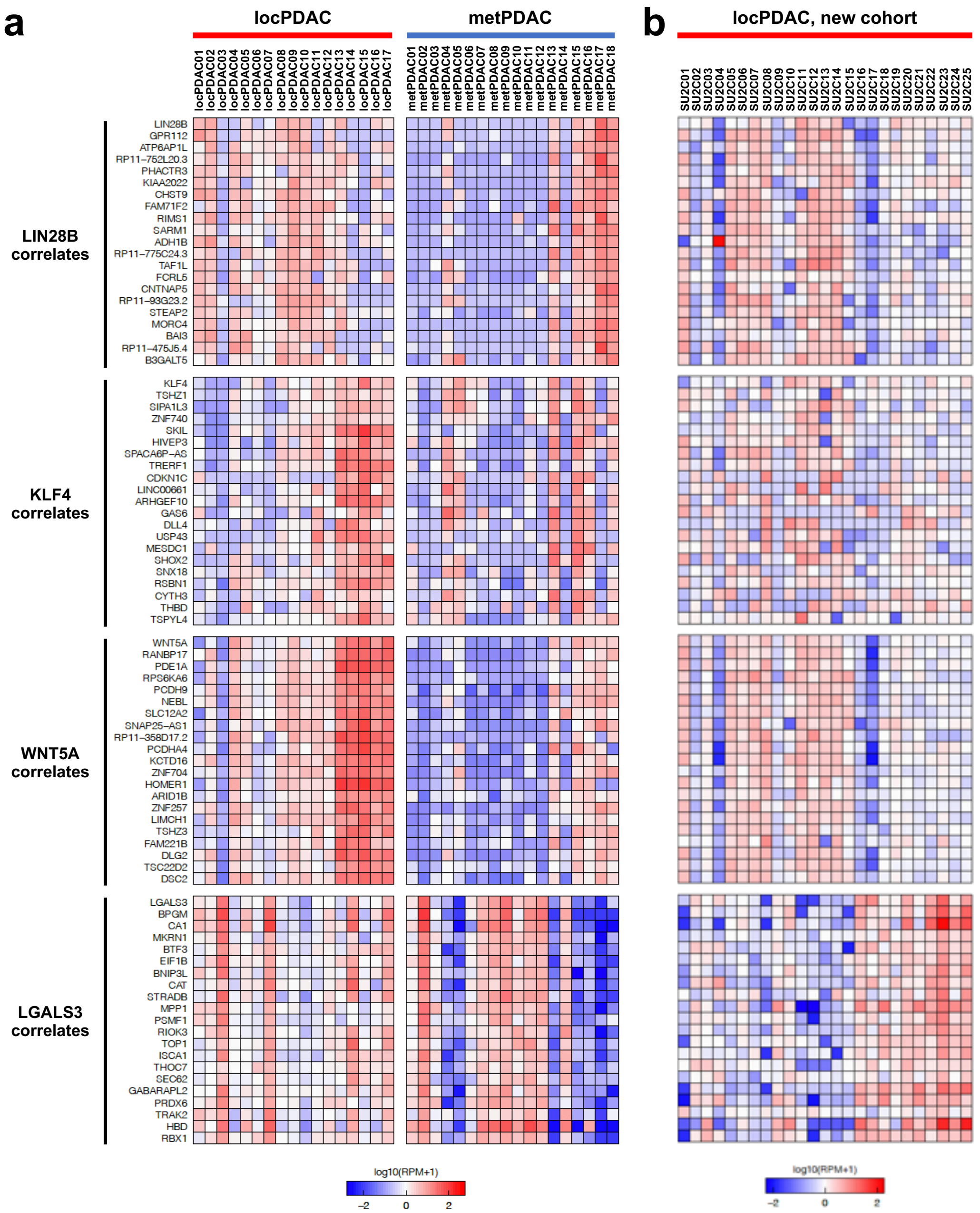

Supplementary Figure 2. Heatmaps of locPDAC cohort, metPDAC cohort, and additional locPDAC cohort. (**a**) Heatmap ($\log_{10}$(RPM+1), median-subtracted) of the *LIN28B*, *KLF4*, *WNT5A*, and *LGALS3* and each of the top 20 genes correlated with each of these genes in the locPDAC and metPDAC dataset. (**b**) Heatmap ($\log_{10}$(RPM+1)) of the same genes as in (A) but for an independent dataset of patients with borderline resectable or locally advanced PDAC.

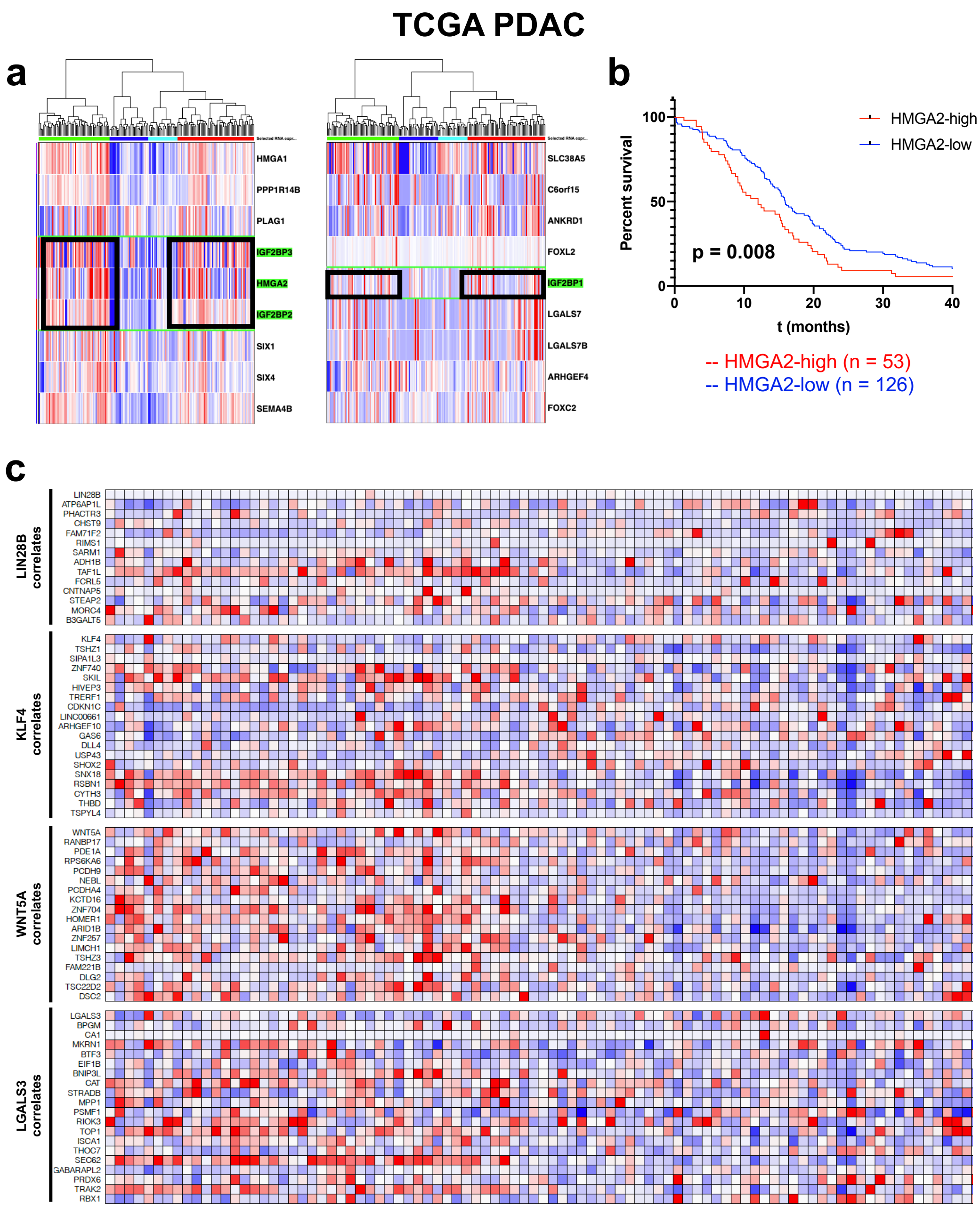

Supplementary Figure 3. LIN28B pathway genes cluster together in resected PDAC tissue (TCGA data). (**a**) *HMGA2* and *IGFBP1/2/3* cluster together in the TCGA resected PDAC tissue dataset. Heat map of Z-score normalized data of 185 PDAC tissue specimens utilizing the Cbioportal clustered heat map tool[8]. (**b**) High *HMGA2* expression correlates with poor outcome in PDAC. * p = 0.008 by Gehan-Breslow-Wilcoxon test, n = 53 in the HMGA2-high group and 126 in the HMGA2-low group. (**c**) Genes that correlate with *LIN28B*, *KLF4*, *WNT5A*, and *LGALS3* in our PDAC CTC dataset do not correlate with each other in resected PDAC tissue.

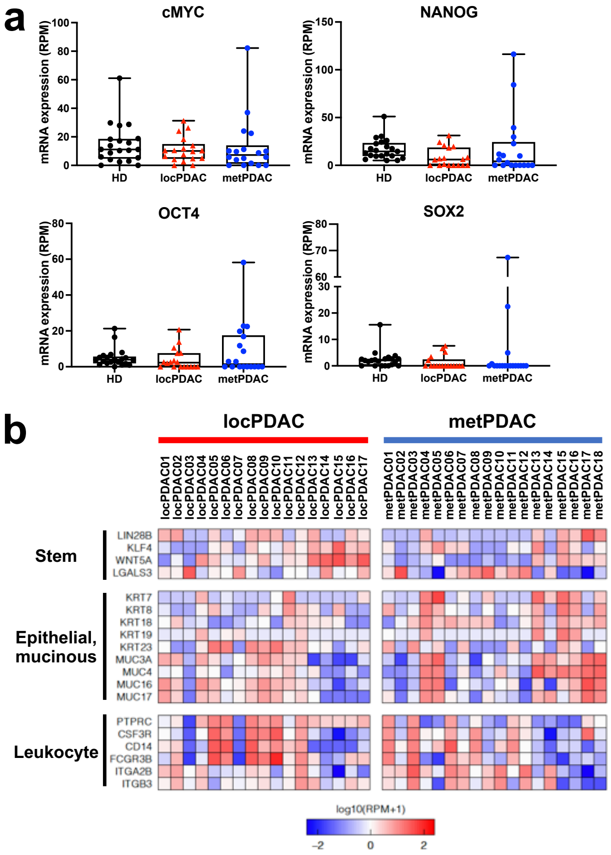

Supplementary Figure 4: Other pluripotency marker mRNAs are not enriched in CTCs purified from the blood of non-metastatic PDAC patients and metastatic PDAC patients. (**a**) Gene expression (in reads-per-million, RPM) boxplots of *cMYC*, *NANOG*, *OCT4*, and *SOX2* in locPDAC and metPDAC CTCs. * FDR >0.1 for all inter-group comparisons (n = 21 HD, 17 locPDAC, 18 metPDAC), box plots show median and 25-75% IQR. (**b**) *LIN28B* expression correlates with mucin gene expression but does not correlate with leukocyte gene expression in our bulk CTC RNA-seq gene expression dataset.

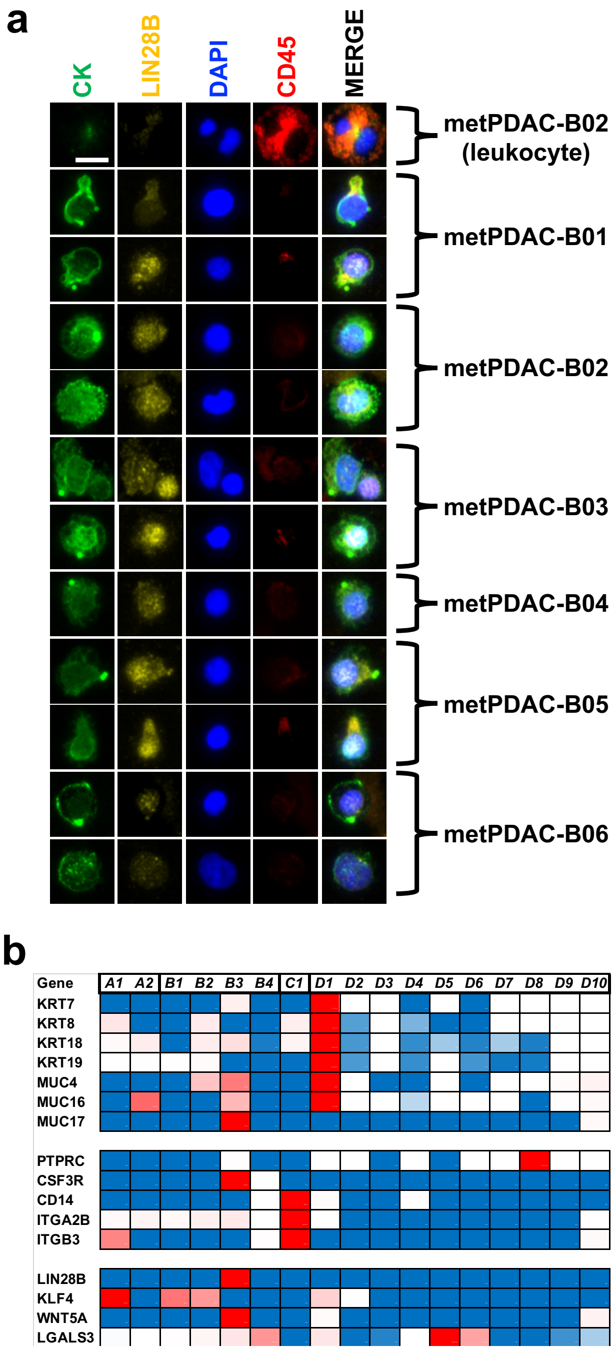

Supplementary Figure 5: Immunofluorescent staining of CTCs from an independent cohort of patients with metastatic PDAC demonstrates heterogeneous nuclear expression of LIN28B. (**a**) Indirect immunofluorescence images of CTCs purified with the CTC-iChip, fixed, spun onto slides, and imaged. Pan-cytokeratin (CK) is pseudocolored in green, LIN28B in yellow, DNA from DAPI staining in blue, and CD45 in red. A CD45+ leukocyte derived from one of the patients is shown in the first "row" as a positive control for CD45 staining. This experiment was not repeated (**b**) Single-cell data from prior work[9], showing 17 micro-manipulated human PDAC CTCs showing expression of *KRT*, *MUC*, leukocyte (*PTPRC*, *CSF3R*, *CD14*, *ITGA2B*, *ITGB3*), and stem cell (*LIN28B*, *KLF4*, *WNT5A*, *LGALS3*) genes. Data are presented in RPM, with red

showing the highest value and blue the lowest value (with white as a central point) in each row/gene.

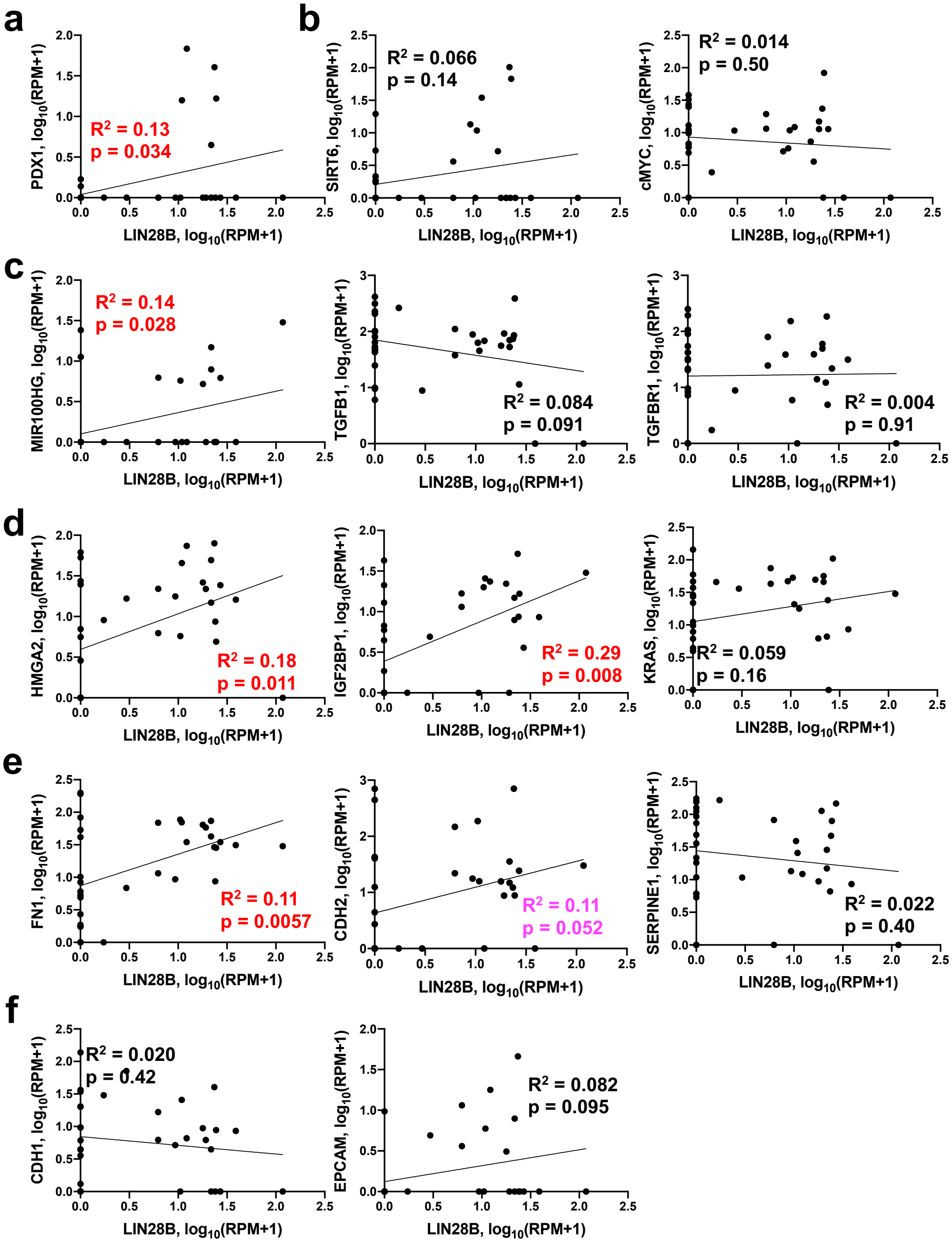

Supplementary Figure 6: **LIN28B expression in PDAC CTCs correlates with several known associated driver genes**. (**a**) LIN28B mRNA expression correlates with PDX1, a known pancreatic transcription factor. (**b**) SIRT6 and cMYC do not correlate with LIN28B expression in PDAC CTCs. (**c**) miR100HG, a lncRNA driven by TGFβ signaling and associated with LIN28B, but not TGFβ1 or TGFβR1, correlates with LIN28B expression in PDAC CTCs. (**d**) LIN28B expression in PDAC CTCs correlates with let-7 targets HMGA2 and IGF2BP1 but not KRAS. (**e**) Quasi-mesenchymal gene FN1, but not SERPINE1, correlate with LIN28B in PDAC CTCs; CDH2 (N-cadherin) trends towards significance. (**f**) Epithelial genes CDH1 (E-cadherin) and

EPCAM do not correlate with LIN28B in PDAC CTCs. * $p < 0.05$ by least squares linear regression, with no adjustments made for multiple tests.

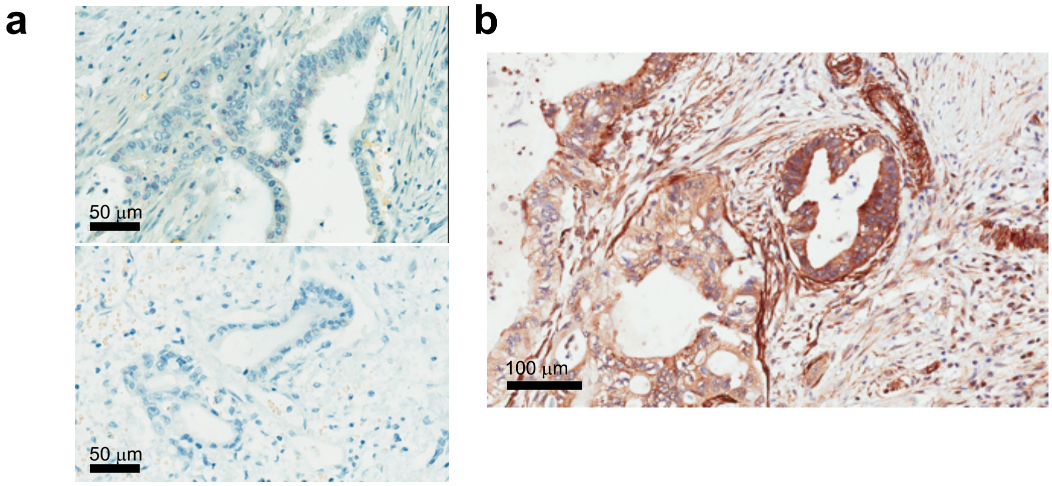

Supplementary Figure 7: LIN28B expression in resected PDAC tissue is heterogeneous. (**a**) RNA in situ hybridization (RNA-ISH) staining of resected PDAC tissue demonstrates “positive” (top micrograph) and “negative” (bottom micrograph) *LIN28B* staining within glandular PDAC cells. (**b**) Immunohistochemistry staining for LIN28B in resected PDAC tissue also demonstrates protein heterogeneity even between different glands within a given specimen. These results were confirmed on at least 5 resection specimens, confirming a range of RNA (a) and protein (b) expression.

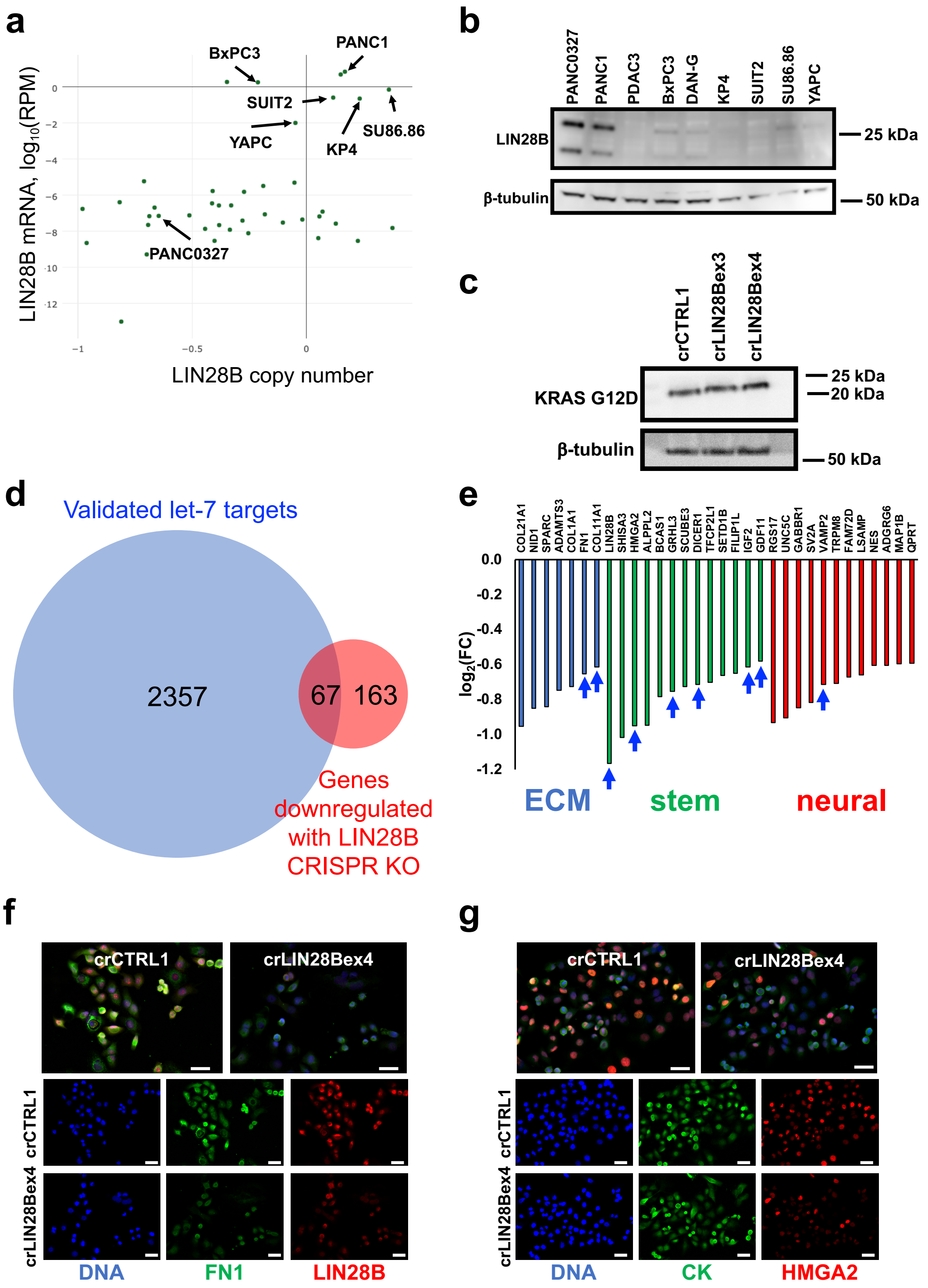

Supplementary Figure 8. Further characterization of the LIN28B phenotype in PANC1 cells. **(a)** Cancer cell line encyclopedia (Broad Institute) data showing variable LIN28B mRNA expression – imperfectly correlated with gene copy number – in well-characterized PDAC cell lines. (**b**) Western blot of PDAC cell lines showing heterogeneous protein expression level of LIN28B in the tested cell lines. These results were verified by one replicate. (**c**) Western blot

showing levels of KRAS G12D (β-tubulin loading control) in PANC1 cells transduced with nonsense CRISPR guides (crCTRL1) or CRISPR guides targeting LIN28B (crLIN28Bex3, crLIN28Bex4). These results were verified by one replicate. **(d)** Venn diagram showing overlap between let-7 targets (validated, per mirTarbase database[7]) and genes downregulated in PANC1 cells upon LIN28B CRISPR knockout. (**e**) Curated subset of 32 of the top 100 downregulated genes (each with FDR <0.10 vs crCTRL1 group) upon LIN28B CRISPR knockout in PANC1 cells grouped by ontology; blue arrows denote known let-7 targets. (**f,g**) Representative immunofluorescent images of LIN28B, FN1, CK8/18, and HMGA2 protein in PANC1 cells transduced with nonsense gRNAs (crCTRL1) or gRNA targeting LIN28B exon 4. Scale bar is 50 μm. These results were verified by one replicate.

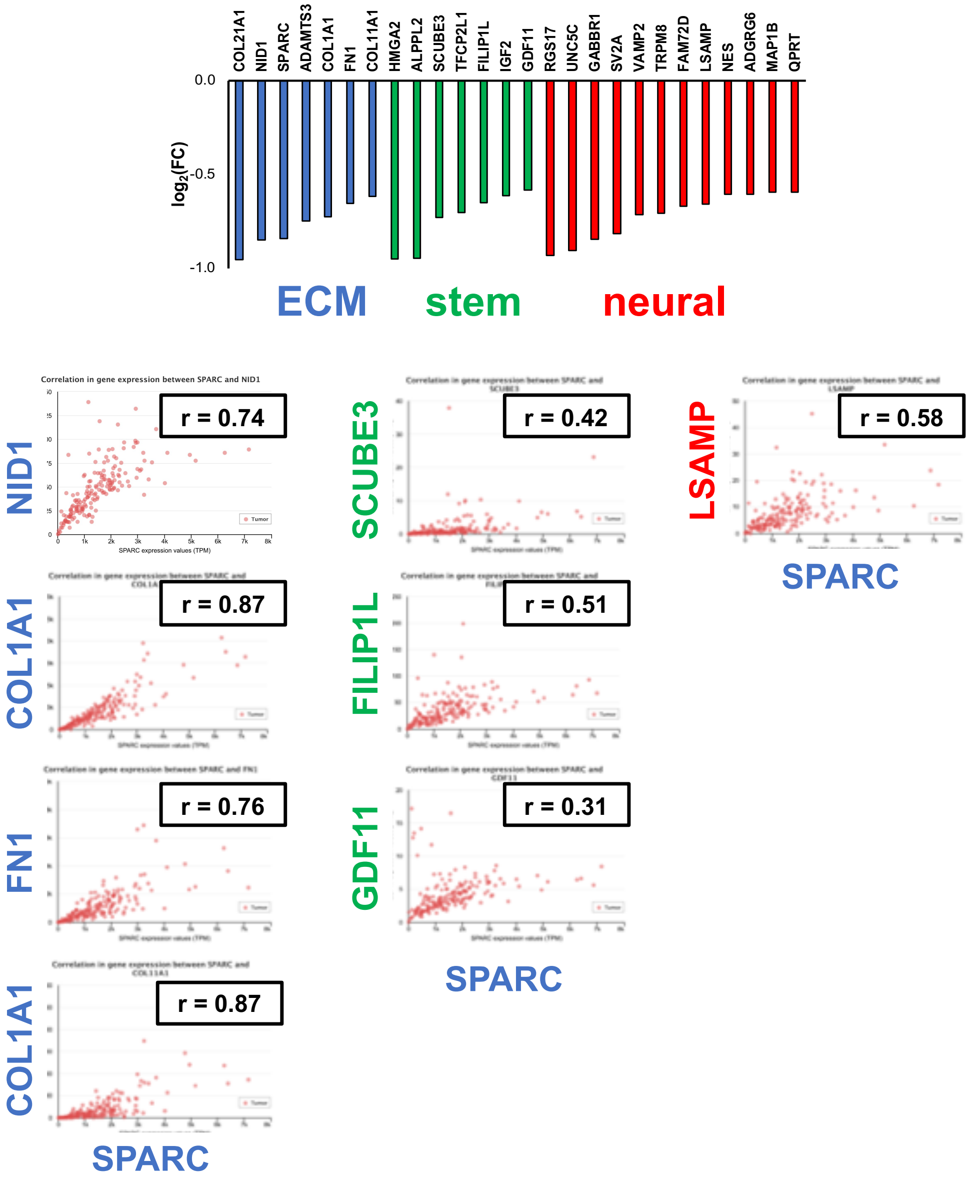

Supplementary Figure 9. Genes that are downregulated upon LIN28B knockout in PANC1 cells are also correlated in human primary PDAC tumours catalogued in the TCGA database. Bar graph reproduced from Supplementary Fig. S8E (Curated subset of 32 of the top 100 downregulated genes (each with FDR <0.10 vs crCTRL1 group) upon LIN28B CRISPR knockout in PANC1 cells grouped by ontology; blue arrows denote known let-7 targets). Pearson correlation coefficients for significantly ($p<0.05$) correlated genes from TCGA primary data were obtained with the UALCAN online tool[10].

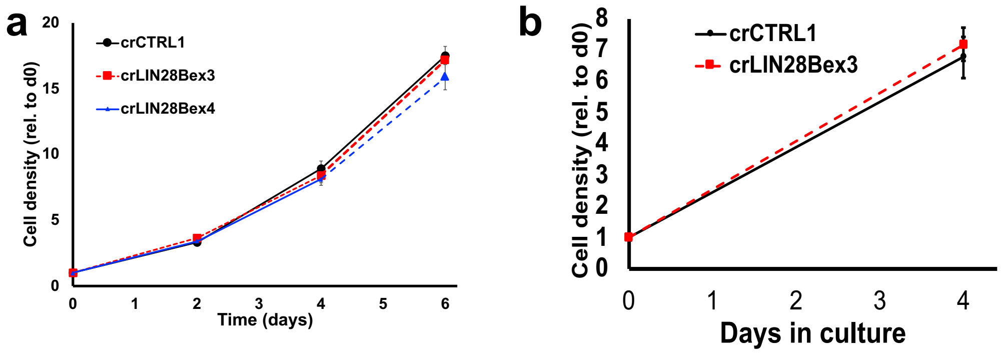

Supplementary Figure 10. LIN28B-knockout PDAC cells proliferate the same as control cells. PANC1 (**a**) and PANC0327 (**b**) cell proliferation kinetics as measured by resazurin reduction, normalized to the signal at day 0. p = N.S. by 2-tailed Bonferonni-adjusted t-test at final experiment timepoint

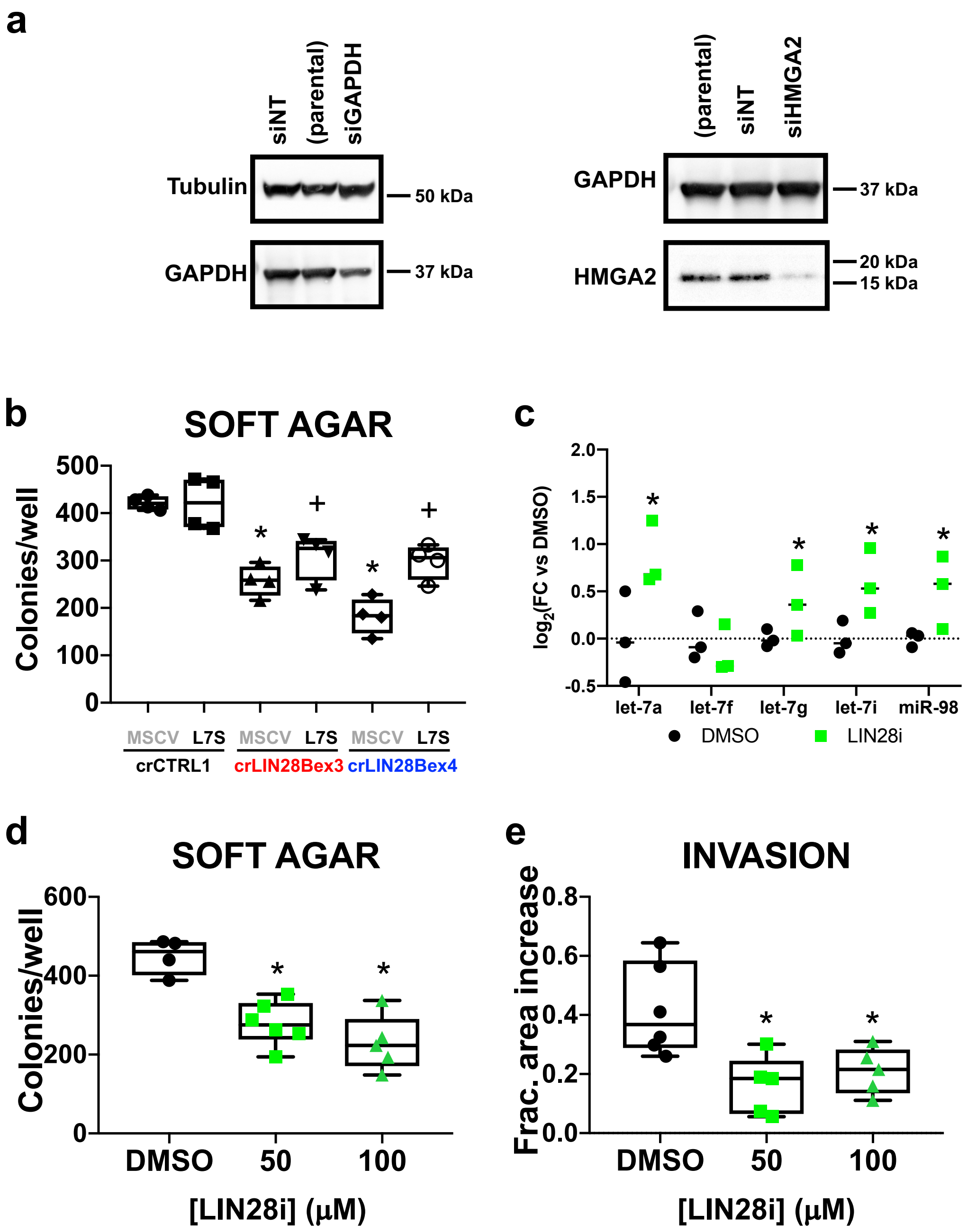

Supplementary Figure 11. The canonical LIN28B/let-7/HMGA2 pathway dictates LIN28B effects on nonadherent phenotype in PANC1 cells. (a) Soft agar colony formation after silencing FN1 or IGF2BP1 (versus transfection with a non-targeting control siRNA, siNT). These results were verified by one replicate. **(b)** Soft agar colony formation after transfection of LIN28B-knockout (crLIN28Bex3, crLIN28Bex4) or control (crCTRL1) PANC1 cells with either empty MSCV-puro plasmid or MSCV-puro plasmid containing a let-7 sponge (L7S). * p<0.001 by Bonferonni-adjust 2-tailed t-test versus control, $^{+}$ p<0.05 versus MSCV puro within the subgroup, n = 4 per group, box plots showing median and 25-75% IQR. **(c)** miRNA qPCR of mature let-7 species after 6 days of treatment with 50 μM LIN28 inhibitor. * p<0.05 by Holm-Sidlak adjusted 2-tailed t-test, n = 3 per group. **(d,e)** Dose-response assay comparing soft agar colony formation

(**D**) and 3D invasion (**e**) of PANC1 cells treated with 50 or 100 μM of a LIN28B inhibitor, with vehicle-treated (DMSO) cells as a control. * $p<0.005$ by Bonferroni-adjusted 2-tailed t-test versus DMSO, n = 4-6 per group, 25-75% IQR shown in box plots.

Supplementary References

1. Indolfi L*, et al.* A tunable delivery platform to provide local chemotherapy for pancreatic ductal adenocarcinoma. *Biomaterials* **93**, 71-82 (2016).

2. Kugel S*, et al.* SIRT6 Suppresses Pancreatic Cancer through Control of Lin28b. *Cell* **165**, 1401-1415 (2016).

3. Ligorio M*, et al.* Stromal Microenvironment Shapes the Intratumoral Architecture of Pancreatic Cancer. *Cell*, (2019).

4. Vinci M, Box C, Eccles SA. Three-dimensional (3D) tumor spheroid invasion assay. *J Vis Exp*, e52686 (2015).

5. Cerami E*, et al.* The cBio cancer genomics portal: an open platform for exploring multidimensional cancer genomics data. *Cancer Discov* **2**, 401-404 (2012).

6. Gao J*, et al.* Integrative analysis of complex cancer genomics and clinical profiles using the cBioPortal. *Sci Signal* **6**, pl1 (2013).

7. Chou CH*, et al.* miRTarBase update 2018: a resource for experimentally validated microRNA-target interactions. *Nucleic Acids Res* **46**, D296-D302 (2018).

8. Ryan MC*, et al.* Interactive Clustered Heat Map Builder: An easy web-based tool for creating sophisticated clustered heat maps. *F1000Res* **8**, (2019).

9. Ting DT*, et al.* Single-cell RNA sequencing identifies extracellular matrix gene expression by pancreatic circulating tumor cells. *Cell Rep* **8**, 1905-1918 (2014).

10. Chandrashekar DS*, et al.* UALCAN: A Portal for Facilitating Tumor Subgroup Gene Expression and Survival Analyses. *Neoplasia* **19**, 649-658 (2017).
