# Supplementary material for: Pancreatic circulating tumor cell profiling identifies LIN28B as a metastasis driver and drug target
Source: Nat Commun. 2020 Jul 3;11:3303. doi: 10.1038/s41467-020-17150-3 (PMC7335061; doi:10.1038/s41467-020-17150-3)
Supplement: Supplementary file 3 — Reporting Summary [file 41467_2020_17150_MOESM3_ESM.pdf]

nature research

Corresponding author(s): David T. Ting

Last updated by author(s): Jun 1, 2020

Reporting Summary

Nature Research wishes to improve the reproducibility of the work that we publish. This form provides structure for consistency and transparency in reporting. For further information on Nature Research policies, see our Editorial Policies and the Editorial Policy Checklist.

Statistics

For all statistical analyses, confirm that the following items are present in the figure legend, table legend, main text, or Methods section.

n/a	Confirmed	
☐	☒	The exact sample size (n) for each experimental group/condition, given as a discrete number and unit of measurement
☐	☒	A statement on whether measurements were taken from distinct samples or whether the same sample was measured repeatedly
☐	☒	The statistical test(s) used AND whether they are one- or two-sided<br>*Only common tests should be described solely by name; describe more complex techniques in the Methods section.*
☒	☐	A description of all covariates tested
☐	☒	A description of any assumptions or corrections, such as tests of normality and adjustment for multiple comparisons
☐	☒	A full description of the statistical parameters including central tendency (e.g. means) or other basic estimates (e.g. regression coefficient) AND variation (e.g. standard deviation) or associated estimates of uncertainty (e.g. confidence intervals)
☐	☒	For null hypothesis testing, the test statistic (e.g. F, t, r) with confidence intervals, effect sizes, degrees of freedom and P value noted<br>*Give P values as exact values whenever suitable.*
☒	☐	For Bayesian analysis, information on the choice of priors and Markov chain Monte Carlo settings
☒	☐	For hierarchical and complex designs, identification of the appropriate level for tests and full reporting of outcomes
☐	☒	Estimates of effect sizes (e.g. Cohen's d, Pearson's r), indicating how they were calculated

Our web collection on statistics for biologists contains articles on many of the points above.

Software and code

Policy information about availability of computer code

Data collection	Rstudio 1.2 and Bioconductor were used for the RNA sequencing analysis . Graphpad Prism software (v8) or Microsoft Excel (v15) was used for statistical testing. Graphpad Prism software (v8) was used for Kaplan-Meier curve generation and analysis.
Data analysis	Differential expression between datasets was performed using the DESEQ2 package in Rstudio. Gene count tables were input to the software then used in a manner similar to that in the sample vignettes in the user manuals. Graphpad prism v8 and Microsoft Excel were used for analysis.

For manuscripts utilizing custom algorithms or software that are central to the research but not yet described in published literature, software must be made available to editors and reviewers. We strongly encourage code deposition in a community repository (e.g. GitHub). See the Nature Research guidelines for submitting code & software for further information.

Data

Policy information about availability of data

All manuscripts must include a data availability statement. This statement should provide the following information, where applicable:

- Accession codes, unique identifiers, or web links for publicly available datasets
- A list of figures that have associated raw data
- A description of any restrictions on data availability

We have added the raw data from the in vitro and animal experiments available as part of the "SourceData.xlsx" file. We deposited the RNA seq data into the Gene Expression Omnibus (GEO), accession number GSE144561. TCGA data were accessed from Cbioportal in February 2020. MirTarBase version 7.0 (http://mirtarbase.mbc.nctu.edu.tw/php/index.php) was used for miRNA target identification.

Field-specific reporting

Please select the one below that is the best fit for your research. If you are not sure, read the appropriate sections before making your selection.

[x] Life sciences [ ] Behavioural & social sciences [ ] Ecological, evolutionary & environmental sciences

For a reference copy of the document with all sections, see nature.com/documents/nr-reporting-summary-flat.pdf

Life sciences study design

All studies must disclose on these points even when the disclosure is negative.

Sample size	Sample sizes for cell culture and in vitro experiments were determined using past experience, usually with between 3 and 6 technical replicates per experiment (see e.g. Ligorio et al, Cell 2020). Sample sizes for animal experiments were also determined using past experience, usually with 6-8 animals per experimental group in a given experiment (also per Ligorio et al, Cell 2020).
Data exclusions	No data were excluded from the analyses
Replication	Each in vitro experiment was replicated at least twice and each replicate was successful. Animal experiments were not replicated but results were concordant. Human studies were not replicated but analysis of human data was performed on an independent cohort of patients and the results were concordant.
Randomization	Allocation between groups was not random and was not relevant to this study, as we were collecting blood from defined cohorts of patients (e.g. patients with localized pancreatic cancer vs patients with metastatic pancreatic cancer) who were receiving clinical care at our hospital.
Blinding	Blinding was not relevant to our study. Analysis required knowledge of which group to which each subject belonged. However, there was no knowledge of patient characteristics by the research staff who was performing the RNA sequencing library preparation.

Reporting for specific materials, systems and methods

We require information from authors about some types of materials, experimental systems and methods used in many studies. Here, indicate whether each material, system or method listed is relevant to your study. If you are not sure if a list item applies to your research, read the appropriate section before selecting a response.

Materials & experimental systems

n/a	Involved in the study	
[ ]	[x]	Antibodies
[ ]	[x]	Eukaryotic cell lines
[x]	[ ]	Palaeontology and archaeology
[ ]	[x]	Animals and other organisms
[ ]	[x]	Human research participants
[ ]	[x]	Clinical data
[ ]	[x]	Dual use research of concern

Methods

n/a	Involved in the study	
[x]	[ ]	ChIP-seq
[x]	[ ]	Flow cytometry
[x]	[ ]	MRI-based neuroimaging

Antibodies

Antibodies used	IHC: IHCPlus™ Polyclonal LIN28B Antibody (LS Bio, Catalog Number LS-B3423-200), diluted to 10ug/ml.<br>IF: FN1 (Santa Cruz Biotechnology, catalog #sc-8422, 1:50 dilution), HMGA2 (Cell Signaling Technology, catalog #8179, 1:200 dilution), LIN28B (Cell Signaling Technology, catalog #11965, 1:50<br>dilution), CK8/18 (Cell Signaling Technology, catalog #4546, 1:200 dilution), pan-keratin (Cell Signaling Technology, catalog #4545 1:500 dilution), CD45 (Cell Signaling Technology, catalog #55307,<br>1:500 dilution).<br>WB: LIN28B (catalog #11965, 1:1000 dilution), tubulin (catalog #2125, 1:5000 dilution), KRAS G12D (catalog #14429, 1:1000 dilution), HMGA2 (catalog #8179, 1:1000 dilution), GAPDH (catalog #5174,<br>1:5000 dilution, Anti-rabbit IgG HRP-conjugated (catalog #7054, 1:1000 dilution), Anti-mouse IgG HRP-conjgated (catalog #7076, 1:1000 dilution<br>Each antibody that was used was used in applications that were validated by the manufacturer per their product information
Validation	Each antibody that was used was used in applications that were validated by the manufacturer per their product information

Eukaryotic cell lines

Policy information about cell lines

Cell line source(s)	Cell lines were obtained from ATCC (Panc1, Panc0327, MiaPaca2, SUIT2, KP4, DAN-G, YAPC, BxPC3, SU86.86, HEK-293T) or generated in our own laboratory (PDAC3). PANC1 and PANC0327 were modified by lentiCRISPRv2 transduction delivery of either nonsense CRISPR guides or guides targeted against LIN28B exons 3 or 4.
Authentication	The cell lines were not authenticated.
Mycoplasma contamination	A fresh aliquot of cells was used and passaged for no more than 1 month of use prior to discarding. Frozen cell stocks were tested every 3-6 months and not found to be contaminated with Mycoplasma.
Commonly misidentified lines (See ICLAC register)	No commonly misidentified cell lines were used in this study.

Animals and other organisms

Policy information about studies involving animals; ARRIVE guidelines recommended for reporting animal research

Laboratory animals	Female NSG (NSG; NOD.Cg-Prkdcscid Il2rgtm1Wjl/Sz)) mice betwen 6-8 weeks of age were purchased from Jackson labs. They were housed in cages of no more than 4 mice/cage at a temperature of 23+/- 3 degree C and relative humidity of 30-70% with 14 hr / 10 hr light/dark cycle.
Wild animals	No wild animals were used.
Field-collected samples	No field-collected samples were used.
Ethics oversight	All animal experiments and animal care were performed according to institutional guidelines at Massachusetts General Hospital (MGH), and approved by the animal protocol (2014N000321).

Note that full information on the approval of the study protocol must also be provided in the manuscript.

Human research participants

Policy information about studies involving human research participants

Population characteristics	Human blood for CTC analysis was obtained on existing Institutional Review Boards protocols (05-300. 18-179) at the Massachusetts General Hospital (MGH). Blood samples from healthy donors were obtained from anonymized discarded specimens collected at a blood donation center. Patients were consented and enrolled prior to blood draws. Pertinent information regarding patient characteristics provided in Tables 1-2 and Supplementary Table 1.
Recruitment	Participants were approached after seeing their treating providers at the Massachusetts General Hospital to obtain consent for additional blood to be drawn periodically when they needed a clinical blood draw. As the patients were not randomized, there is the potential for selection bias, and this was not controlled for in the present study.
Ethics oversight	The protocol has been approved by the MGH and Dana-Farber Harvard Cancer Center (DFHCC) IRBs. Specifically the blood samples were collected under pre-existing protocols DFHCC 05-300 and DFHCC 18-179.

Note that full information on the approval of the study protocol must also be provided in the manuscript.

Clinical data

Policy information about clinical studies

All manuscripts should comply with the ICMJEguidelines for publication of clinical research and a completedCONSORT checklist must be included with all submissions.

Clinical trial registration	*Provide the trial registration number from ClinicalTrials.gov or an equivalent agency.*
Study protocol	*Note where the full trial protocol can be accessed OR if not available, explain why.*
Data collection	*Describe the settings and locales of data collection, noting the time periods of recruitment and data collection.*
Outcomes	*Describe how you pre-defined primary and secondary outcome measures and how you assessed these measures.*

Dual use research of concern

Policy information about dual use research of concern

Hazards

Could the accidental, deliberate or reckless misuse of agents or technologies generated in the work, or the application of information presented in the manuscript, pose a threat to:

No	Yes	
☐	☐	Public health
☐	☐	National security
☐	☐	Crops and/or livestock
☐	☐	Ecosystems
☐	☐	Any other significant area

Experiments of concern

Does the work involve any of these experiments of concern:

No	Yes	
☐	☐	Demonstrate how to render a vaccine ineffective
☐	☐	Confer resistance to therapeutically useful antibiotics or antiviral agents
☐	☐	Enhance the virulence of a pathogen or render a nonpathogen virulent
☐	☐	Increase transmissibility of a pathogen
☐	☐	Alter the host range of a pathogen
☐	☐	Enable evasion of diagnostic/detection modalities
☐	☐	Enable the weaponization of a biological agent or toxin
☐	☐	Any other potentially harmful combination of experiments and agents
